# Editorial: Hepatocellular Carcinoma: From Basic Research to Clinical Trials

**DOI:** 10.3389/fonc.2022.905654

**Published:** 2022-06-01

**Authors:** Xing-Xing He, Da-Wei Ye

**Affiliations:** ^1^ Institute of Liver and Gastrointestinal Diseases, Tongji Hospital, Tongji Medical College, Huazhong University of Science and Technology, Wuhan, China; ^2^ Department of Oncology, Tongji Hospital of Tongji Medical College, Huazhong University of Science and Technology, Wuhan, China

**Keywords:** hepatocellular carcinoma, epigenetic alteration, targeted therapy, diagnosis, prognosis, bioinformatic analysis

Recently, with the rapid advancement of molecular biology techniques, such as sequencing, microarrays, and various omics, a comprehensive and better understanding of hepatocellular carcinoma (HCC) has been acquired, and major breakthroughs have been made in the clinical trials of new drug development in HCC (1).

In this Research Topic, we compiled a series of high-quality papers that summarized recent developments of molecular mechanism, diagnosis, treatment, and prevention of HCC. The topic starts with a number of reviews and research articles that form an update of epigenetic alteration knowledges in HCC. As one of the epigenetic factors involved in the pathogenesis of HCC, long non-coding RNAs (lncRNAs) were found to be frequently dysregulated. Ghafouri-Fard et al. summarized the recent finding of lncRNAs which contributed in the pathogenesis of HCC, and Xia et al. constructed a model based on glycolysis-related lncRNAs to reflect the prognosis of HCC patients. Besides, Zhang and Wang described the biogenesis, categories, and functions of circRNAs, as well as reviewed the crucial role of circRNAs as potential biomarkers and therapeutic targets in HCC. Moreover, Hong et al. investigated the mechanism of miR-21-3p promoting migration and invasion of HCC cells. Dong et al. focus on the importance of aberrant methylation status in the precision usage of alternative promoters in HCC, a way contributes to the cellular transformation of cancer.

The Research Topic goes on to the discussion of integrated multi-omics studies in HCC. Lin et al. identified a signature of eight inflammatory response-related genes by combining transcriptome with proteomic analyses, which is useful in prognostic prediction and influencing the immune status in HCC.

The Research Topic also pays attention to the diagnosis and prognosis of HCC. Yang et al. reviewed the role of liquid biopsy as a novel clinical biomarker for diagnosis of HCC, including but not limited to exosome in early diagnosis, prognostic evaluation, disease surveillance, and instructing treatment. In addition, Si et al. found that CCNB1, CDC20, and CENPF genes were frequently upregulated, and these genes exhibited great potential value in early diagnosis and prognosis for HCC patients. Furthermore, Hu et al. initiated an original and effective nomogram for predicting the individual probability of recurrence after PA-TACE, and identified which kind of patients may benefit from PA-TACE after surgery.

Finally, the Research Topic collected a series of high-quality papers about the treatment of HCC, covering the exploration and discussion of various treatment methods. Niu et al. introduced targeted agents with their clinical efficacy for advanced HCC, discussed the hopeful targets for drug development, and showed the effective outcomes when adopting targeted therapy in combination with immune checkpoint inhibitors. Shao et al. discovered that the combination of Crizotinib and doxorubicin (Dox) can effectively reduce Dox resistance and promote liver cancer cell death by reducing multidrug-resistant 1 (MDR1) protein. Nath et al. found that the chemotherapy effect of UTT-B, a saponin extracted from the leaves of Solanum nigrum, against HCC can be further enhanced by blocking autophagy pro-survival signal, and can also be enhanced by combination with Chloroquine. Besides, Zhu et al. aimed at the role of ubiquitin-mediated degradation of related genes in the tumorigenesis of HCC, and found it hold great promise in targeting the TRIM54/Axin1/β-catenin axis for HCC prognosis and treatment.

The editors thank all reviewers and authors for their valuable contributions. We wish that this Research Topic would inspire more discussions and thoughts for future HCC study.

## Author Contributions

All authors listed have made a substantial, direct, and intellectual contribution to the work and approved it for publication.

## Funding

This research was financially supported by the National Natural Science Foundation of China (No. 82073095).

## Conflict of Interest

The authors declare that the research was conducted in the absence of any commercial or financial relationships that could be construed as a potential conflict of interest.

## Publisher’s Note

All claims expressed in this article are solely those of the authors and do not necessarily represent those of their affiliated organizations, or those of the publisher, the editors and the reviewers. Any product that may be evaluated in this article, or claim that may be made by its manufacturer, is not guaranteed or endorsed by the publisher.
